# Unravelling the Role of miR-20b-5p, CCNB1, HMGA2 and E2F7 in Development and Progression of Non-Small Cell Lung Cancer (NSCLC)

**DOI:** 10.3390/biology9080201

**Published:** 2020-08-01

**Authors:** Shweta Arora, Prithvi Singh, Arshad Husain Rahmani, Saleh A. Almatroodi, Ravins Dohare, Mansoor Ali Syed

**Affiliations:** 1 Translational Research Lab, Department of Biotechnology, Faculty of Natural Sciences, Jamia Millia Islamia, New Delhi 110025, India; shweta169213@st.jmi.ac.in; 2 Centre for Interdisciplinary Research in Basic Sciences, Jamia Millia Islamia, New Delhi 110025, India; prithvi.mastermind@gmail.com; 3 Department of Medical Laboratories, College of Applied Medical Sciences, Qassim University, Buraydah 51452, Saudi Arabia; ah.rahmani@qu.edu.sa (A.H.R.); smtrody@qu.edu.sa (S.A.A.)

**Keywords:** NSCLC, eigengene, feed-forward loop, prognosis, module

## Abstract

Lung cancer is a prime cause of worldwide cancer deaths, with non-small cell lung cancer (NSCLC) as a frequent subtype. Surgical resection, chemotherapy are the currently used treatment methods. Delayed detection, poor prognosis, tumor heterogeneity, and chemoresistance make them relatively ineffective. Genomic medicine is a budding aspect of cancer therapeutics, where miRNAs are impressively involved. miRNAs are short ncRNAs that bind to 3′UTR of target mRNA, causing its degradation or translational repression to regulate gene expression. This study aims to identify important miRNA-mRNA-TF interactions in NSCLC using bioinformatics analysis. GEO datasets containing mRNA expression data of NSCLC were used to determine differentially expressed genes (DEGs), and identification of hub genes-BIRC5, CCNB1, KIF11, KIF20A, and KIF4A (all functionally enriched in cell cycle). The FFL network involved, comprised of miR-20b-5p, CCNB1, HMGA2, and E2F7. KM survival analysis determines that these components may be effective prognostic biomarkers and would be a new contemplation in NSCLC therapeutics as they target cell cycle and immunosurveillance mechanisms via HMGA2 and E2F7. They provide survival advantage and evasion of host immune response (via downregulation of cytokines-IL6, IL1R1 and upregulation of chemokines-CXCL13, CXCL14) to NSCLC. The study has provided innovative targets, but further validation is needed to confirm the proposed mechanism.

## 1. Introduction

Lung cancer is the trivial cause of cancer-associated worldwide deaths with a flat overall 5-year survival rate of less than 15%. It is patho-physiologically divided into two subgroups: highly aggressive, but less frequent (~15%) small cell lung cancer (SCLC) and less aggressive but highly intermittent (~85%) non-small cell lung cancer (NSCLC). NSCLC is further histologically subdivided into three subtypes—adenocarcinoma (40%), squamous cell carcinoma (25%), and large cell carcinoma (10%). Lung cancer usually commences with oncogene activation or tumor suppressor gene inactivation [1]. Despite developments of innovative therapies, the survival rate for NSCLC patients still remains low. Late disease presentation, histological subtype tumor heterogeneities, and restrained understanding of tumor biology are the key causes of poor prognosis. Distinguished pathological and histological phenotypic characterization of LC has allowed us to discriminate NSCLC and SCLC as two different diseases. Nonetheless, recent clinical observations have proposed that NSCLC to SCLC transformations may exist. Inhibition of epidermal growth factor receptor (EGFR) has been partly accredited to this transformation [2].

Non-metastatic lung tumors present in early stages are subjected to surgical resection for treatment but advanced or metastatic lung cancer requires chemotherapy, either alone or in concert with radiation [3]. However, the competence of chemotherapy has been volatile and awfully compressed because of inherent or acquired drug resistance. Several mechanisms such as drug target modifications, deregulated apoptosis, enhanced drug efflux, and activation of alternative survival signaling cascades are responsible for the development of drug resistance. Moreover, NSCLC therapy has reached a plateau phase because of restrained perception of pathogenesis and fluctuating gene expression profiles of tumors. Therefore, genomic medicine is an emerging area in this direction that would compensate investigations into lung cancer oncogenesis. It would be a new contemplation in development of molecular diagnosis, new biomarkers, and risk stratification of lung cancer (my paper). Only 2% of the human genome encodes for protein-coding genes, however there is an extensive preponderance of transcripts that are non-coding RNAs, inclusive of microRNAs (miRNAs) [4].

miRNAs are non-coding RNAs, which are very short ~20–24 nucleotides in length [5]. miRNAs are defined as such on the basis of the activity of an enzyme dicer, (an RNAse involved in the processing of hairpin structure pre-miRNA into mature miRNAs), involved in their biogenesis [6,7]. They recognize and bind to the complementary target sites in the 3′UTR (3′untranslated region) of target mRNA resulting in post-transcriptional gene silencing [8]. Such repression of genes is mediated by mature mi-RNAs and Argonaute (AGO) proteins, a constituent of RNA-induced silencing complex (RISC) [9]. As a consequence, it induces mRNA degeneration or translational inhibition depending upon the level of complementarity between miRNA and 3′UTR of target sites. They are known to modulate 20–30% of all human transcripts, therefore being involved in almost all signaling pathways [10]. Thus, dysregulation of miRNA may lead to enormous pathological circumstances. It has been found that thousands of miRNAs are associated with various human diseases. There are several lung cancer-related pathways in which miRNAs are inculcated. Signal cascades leading to the synthesis of DNA and cellular proliferation involving receptor tyrosine kinases such as ErbB2 (HER2/Neu), ErbB3, and ErbB4 are implicated in cancer cell proliferation and tumorigenesis [11]. Tumor invasion, angiogenesis, cellular proliferation, and apoptosis is controlled by epidermal growth factor receptor (EGFR)/ErbB1 in NSCLC [1]. For instance, Let-7/miR-98 family of miRNAs target both RAS and MYC and their overexpression and amplification has been observed in varied histologic subtypes of lung cancer. Let-7 family inhibits the expression of a number of oncogenes such as RAS, MYC, and HMGA2 [12,13,14,15]. miR-29 is negatively regulated in lung cancer and has a role in regulating epigenetic DNA methylation in repressing Mcl-1 [16,17,18].

Lung cancer usually occurs as a result of oncogene activation or tumor suppressor gene inactivation. However, 60–75% of NSCLC cases are due to inhibition of tumor suppressor genes such as p53 and Rb. miRNAs play crucial roles in these processes. Other causes include epigenetic modifications, genetic loss, and widespread transcriptional repression, all of which are also associated with aberrant regulation of miRNA levels in lung cancer. miRNAs are also known to exert transcription repression effects mainly by inhibiting transcription factors (TFs). However, there are very few miRNAs that activate transcription. Thus, miRNAs are known to interact with both mRNAs of genes as well as transcription factors (my papers). A thorough consideration of such interactions would be highly beneficial in the development of novel therapeutic strategies.

This study is based on the identification of representative module genes from a scale-free weighted gene co-expression network (GCN) followed by protein–protein interaction (PPI) cluster detection, enrichment analysis, and construction of an FFL network that could probably explore some of the conducive aspects involved in the development of NSCLC. The prognostic significance of associated mRNAs, miRNA and TFs was determined by using the data from The Cancer Genomic Atlas (TCGA) Genotype-Tissue Expression (GTEx) datasets. Genetic modification analysis of hub genes and TFs was done to identify the role of genetic alterations in their aberrant expression. Overall, we have postulated the identification of certain important links and novel prognostic biomarkers through this study. These links, once explored meticulously, could prove to be very useful therapeutic targets for NSCLC.

## 2. Materials and Methods

### 2.1. NSCLC-Associated Microarray Data Extraction

NCBI-Gene Expression Omnibus (GEO) [19] was searched exhaustively for the retrieval of NSCLC-associated mRNA profiles. GEO was queried using “Non-Small Cell Lung Cancer” and “NSCLC” as the suitable keywords. Our search criteria for selecting the mRNA datasets were as follows: (1) The datasets must be derived from tumor and adjacent non-tumor tissue of patients with NSCLC (paired samples); (2) datasets must be from Homo sapiens and expression profiling by array type; (3) datasets must be derived from the same microarray platform. Series Matrix expression files of the selected mRNA datasets were downloaded, and any non-paired tissue samples were removed to maintain uniformity in our analysis. Probe IDs were mapped to their corresponding HUGO Gene Nomenclature Committee (HGNC) gene symbol(s) using the GPL570 annotation file and hgu133plus2.db package available in R v4.0.2. Expression values of duplicate gene symbols mapping to multiple probe IDs were averaged across the samples [20,21] in both the datasets followed by *p*-value computation of all unique genes using two-sample paired t-test function. Common genes from both the datasets were identified using the *intersect* function. Meta-analysis was performed by combining the *p*-values of common genes using Fisher’s combined probability test method followed by their False Discovery Rate (FDR) adjustment using Benjamini and Hochberg (BH) correction method [22]. Also, $\log_{2}$ fold change ($\log_{2}\mathrm{FC}$) of all these genes were computed [23]. The meta-differentially expressed genes (meta-DEGs) were screened considering BH-*p*-value < 0.0001 and an absolute $\left|\log_{2}\mathrm{FC}\right|>1.5$ as the preferred threshold. Also, meta-DEGs with $\log_{2}\mathrm{FC}>1.5$ and $\log_{2}\mathrm{FC}<-1.5$ were classified as up and downregulated, respectively.

### 2.2. Weighted GCN Construction and Module Detection

The Weighted Gene Co-expression Network Analysis (WGCNA) package [24] available in R was used for GCN construction and representative module genes identification. The NSCLC-associated DEGs were primarily tested with *goodSamplesGenes* function to remove any unqualified genes and samples. Specifically, for every pair of genes i and j, the absolute value of Pearson correlation is defined as:

(1) $$s_{\mathrm{ij}}=\left|\mathrm{cor}\left(i,j\right)\right|$$

The similarity matrix S is defined as $S=\left[s_{\mathrm{ij}}\right]$. Next, the *pickSoftThreshold* function was used for selecting an appropriate soft-thresholding power (β) based on a scale-free topology criterion [25]. We define a power adjacency function which assists to transform the similarity matrix $\left(s_{\mathrm{ij}}\right)$ into a weighted adjacency matrix $\left(a_{\mathrm{ij}}\right)$.

(2) $$a_{\mathrm{ij}}=\mathrm{power}\left(s_{\mathrm{ij}},\beta \right)\equiv {\left|s_{\mathrm{ij}}\right|}^{\beta }$$

The adjacency matrix was then transformed into a similarity measure topological overlap matrix (TOM) followed by the computation of a TOM-based dissimilarity measure (dissTOM). To group genes with similar patterns of expression across samples, a hierarchical clustering dendrogram using *hclust* function was constructed according to the dissTOM measure. The dynamic tree cut algorithm was applied to detect tightly connected modules of genes from the branches of the tree. A summary profile for each module was computed in the form of their Module Eigengenes (MEs) followed by the dissimilarity of module eigengenes (MEdiss) computation. ME can be regarded as the highest representative gene expression profile of the module. Modules with very similar expression profiles were merged (since their genes were highly co-expressed) by cutting the module eigengene dendrogram at suitable MEdiss threshold. Intramodular connectivity is a quantitative measure of network connectivity with respect to nodes or genes of a specific module. Standard and TOM-based-intramodular connectivity are abbreviated by k.in and ω.in, respectively.

(3) $$k.\mathrm{in}=\overset{n}{\underset{j=1}{\sum }}a_{\mathrm{ij}}$$

(4) $$\omega .\mathrm{in}=\overset{n}{\underset{j=1}{\sum }}\omega_{\mathrm{ij}}$$

where $\omega_{\mathrm{ij}}$ is the topological overlap between i and j nodes. WGCNA function, signedKME was used to compute the module membership (MM) which correlated the gene expression values with ME.

(5) $$\mathrm{kME}\left(i\right)=\mathrm{cor}\left(x_{i},\mathrm{ME}\right)$$

The genes that infirmly correlated to all MEs (|kME|<0.7), were assigned to none of the modules. A gene is vital in the given module if the MM was highly linked to k.in. Important genes within a module are characterized by high k.in and MM. These genes are highly connected with other genes and have a high functional relevance. The intramodular connectivity is computed for each module gene by using the WGCNA function intramodularConnectivity. This function calculates the within module connectivity (k.in), whole network connectivity (kTotal), kOut = kTotal − k.in, and kDiff = k.in − kOut. The genes from each module with MM > 0.9 were considered as the representative genes of modules [26].

### 2.3. PPI Network Construction, Enrichment Analysis, and Hub Genes Identification

The highly representative module genes were used for analyzing the strongest possible interactions among them in the form of PPI network. These genes were imported into the Search Tool for the Retrieval of Interacting Genes (STRING, https://string-db.org/) v11.0 database [27] and an overall score > 0.9 (corresponding to highest confidence) was set as the preferred threshold for the construction of PPI network. The network was subsequently visualized using Cytoscape v3.8.0 [28]. Also, the PPI network was analyzed using Molecular Complex Detection (MCODE) plugin available in Cytoscape to identify densely correlated local regions/clusters. The parameters set in MCODE for cluster detection were as follows: “Degree cutoff = 2”, “node score cutoff = 0.2”, “k-score = 2”, “max. depth = 100”, and “cut style = haircut” [29]. The top-scoring PPI cluster genes were further subjected to Gene Ontology (GO) term and pathway enrichment analysis to identify the hub genes. GO term enrichment analysis was performed using Enrichr [30] gene set libraries like GO Biological Process (BP), GO Molecular Function (MF), and GO Cellular Compartment (CC). Also, the pathway enrichment analysis was performed using different Enrichr gene set libraries like WikiPathways, BioPlanet, and KEGG. To account for multiple comparisons, the pathways and GO terms with a BH-*p*-value < 0.001 were considered as significantly enriched. The common genes in all significantly enriched GO terms and pathways were considered as the hub genes, respectively.

### 2.4. Survival Analysis and Hub Genes Validation

To analyze the prognostic impacts of NSCLC-associated hub genes, Kaplan Meier (KM) plotter database (https://kmplot.com/analysis/) [31] was accessed. This database includes gene expression data, overall survival (OS) information of patients and relapse information corresponding to 21 cancer types from sources like GEO, TCGA, and EGA, respectively. The samples were split into low and high expression cohorts by the median expression value of each hub gene to assess the OS of NSCLC patients. Each hub gene was assigned an Affy ID followed by the removal of outlier arrays to produce the corresponding KM survival plots. Moreover, information on 95% confidence interval (95% CI) with hazard ratio (HR), number-at-risk, and log-rank *p* values were computed and showed on the plot. The genes with log rank *p* < 0.05 were considered as statistically significant. The Gene Expression Profiling Interactive Analysis v2 (GEPIA 2) web-based tool (http://gepia2.cancer-pku.cn/) [32] was accessed for validating the hub genes by comparing their relative expression between normal and NSCLC tissue samples from The Cancer Genome Atlas (TCGA) and Genotype-Tissue Expression (GTEx) databases, respectively. The settings used for boxplot comparison were as follows: $\left|{\mathrm{Log}}_{2}\mathrm{FC}\right|$ cutoff = 1.5, *p*-value cutoff = 0.0001, and jitter size = 0.3. Pathological stage plot analysis was also performed with GEPIA 2 to assess the expression levels of hub genes with respect to NSCLC TNM stages.

### 2.5. Construction of the NSCLC-Specific 3-Node miRNA FFL

The human miRNAs intended to target our hub genes were obtained from miRWalk v3.0 [33] and StarBase v2.0 [34] databases, respectively. miRNAs having a total score > 0.95 and binding region = 3′UTRwere chosen from miRWalk. Highly significant TFs with an integrated top rank score (*p*-value) < 0.001 intended to regulate our hub genes were obtained from ChEAv3.0 database [35]. The miRNAs targeting these TFs were also obtained from miRWalk and StarBase databases with previously described thresholds. A two-tier validation screening was performed to fetch highly confident interaction pairs. In the first-tier, all the obtained miRNAs and TFs were exhaustively searched via available literature studies and the ones having an association with NSCLC were promoted to second-tier. In the second-tier, we checked which of our first-tier miRNAs were evolutionarily conserved in humans and mouse. We obtained mouse miRNAs targeting our hub genes from the same databases (miRWalk and StarBase) with previously described thresholds and the common mouse and first-tier screened miRNAs were considered as highly conserved and confident miRNAs. All the three interaction pairs (i.e., miRNA-gene, TF-gene, and miRNA-TF) were then altered with respect to the first and second-tier validated regulatory elements (i.e., TFs and miRNAs) and merged to construct a 3-node NSCLC-specific miRNA feed-forward loop (FFL) network which was finally visualized using Cytoscape. Highest-order network motif in our network was also identified based on the degree of molecular interactions. The survival and expression analysis of the regulatory items in our network motif (i.e., miRNA and TF) were performed using KM plotter, OncoLnc (http://www.oncolnc.org/), and GEPIA 2, respectively.

### 2.6. Exploratory Genomic Analysis of Hub Genes and TFs

The cBioPortal for Cancer Genomics (https://www.cbioportal.org/) [36] was queried for investigating the alteration frequencies in our hub genes. Pan-lung cancer (TCGA, Nat Genet 2016) dataset [37] was selected in cBioPortal to perform our analysis.

## 3. Results

### 3.1. NSCLC-Associated Meta-DEGs Identification

**Figure 1:**
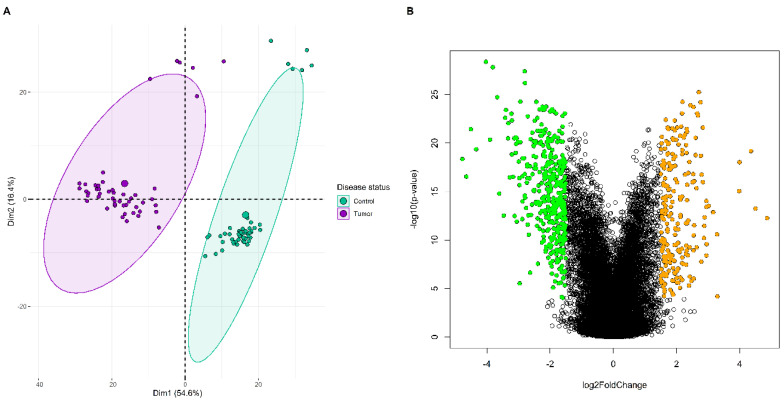
(**A**) Principal component analysis (PCA) plot showing the expression distribution of 603 meta-differentially expressed genes (meta-DEGs) between normal and tumor samples. Every point in the plot represents the relative expression value of all DEGs in each sample. The disease status is distinguished by the color of points, i.e., magenta for tumor and green for normal samples. It could be seen that both the normal and tumor samples are clustered independently and distinctly. The percentage of total variation that is accounted for by the 1st and 2nd principal components are shown on the x and y axes, respectively. (**B**) Volcano plot distribution highlighting 603 meta-DEGs between normal and tumor samples. The orange and green colored points denote the up (218) and downregulated (385) meta-DEGs. All the black colored points denotes non-significant genes. The x and y axes represent the $\log_{2}\mathrm{FC}$ and $-\log_{10}$ *p*-value, respectively.

mRNA datasets with accession numbers GSE118370 and GSE18842 were chosen from GEO based on the search criteria specified in materials and methods section. Both the datasets were based on GPL570 [HG-U133_Plus_2] Affymetrix Human Genome U133 2.0 Array platform type. We considered a total of 100 paired tissue samples (12 from GSE118370 and 88 from GSE18842) for meta-analysis, respectively. There were a total of 20843 unique genes mapping to Affymetrix probes in both datasets. A total of 603 meta-DEGs were identified based on the BH- *p*-value and $\log_{2}\mathrm{FC}$ threshold criterion. A 2-dimensional principal component analysis (PCA) plot representing the variation in expression data of meta-DEGs between tumor and normal samples is shown in Figure 1. Also, a total of 218 and 385 meta-DEGs were classified as up and downregulated based on the $\log_{2}\mathrm{FC}$ threshold criterion and is shown by a volcano plot in Figure 1. List of all DEGs can be found in Table S1.

### 3.2. Weighted GCN Construction and Module Analysis

**Figure 2:**
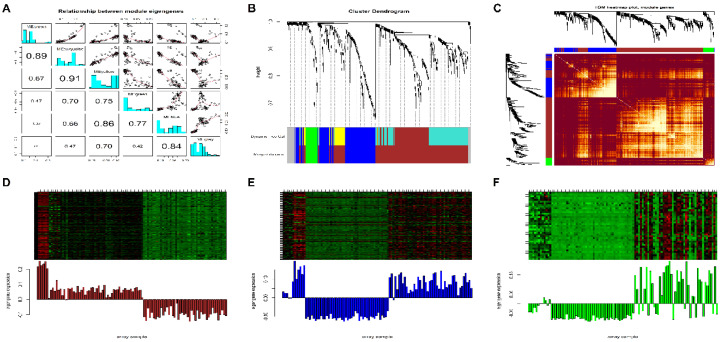
(**A**) Pairwise scatter plots among the MEs of original modules and samples (arrays). Each dot signifies a microarray sample. MEbrown, MEturquoise, MEyellow, MEgreen, MEblue, and MEgrey denote the ME of brown, turquoise, yellow, green, blue, and grey modules, respectively. Absolute values of corresponding correlations are denoted by the numbers below the diagonal. Frequency plots (histograms) of the variables are plotted along the diagonal. Yellow and turquoise modules are highly correlated (i.e., r = 0.91) followed by brown and turquoise modules (i.e., r = 0.89). Both turquoise and yellow modules were merged to the brown module. (**B**) Hierarchical clustering dendrogram of DEGs clustered based on a dissimilarity measure (1-TOM) together with original module colors (6) and merged module colors (4). The four merged modules (or meta-modules) contained highly co-expressed genes with size as follows: brown (361 DEGs), blue (163 DEGs), green (39 DEGs), and grey (40 DEGs) respectively. (**C**) Topological overlap matrix (TOM) heatmap plot where the rows and columns correspond to single genes. Lighter color signifies low topological overlap and progressively darker orange and red colors signify higher topological overlap. Dark colored blocks along the diagonal signify the meta-modules. The corresponding gene dendrogram and module assignment are also displayed along the left and the top sides of the plot. Expression heatmap of (**D**) brown, (**E**) blue, and (**F**) green meta-module genes where the rows and columns correspond to genes and samples, respectively. The green and red colored bands in the heatmap denote lower and higher expression level of genes for each module. Also, the corresponding module eigengene expression levels (y-axis) across the same samples (x-axis) are displayed at the bottom panel of each module heatmap in the form of barplot. The module eigengene levels are directly correlated with the gene expression levels of each corresponding module.

WGCNA was used for categorizing 603 meta-DEGs with similar expression levels into different modules. In our study, we selected β = 16 (at scale free $R^{2}=0.80$) as the soft-thresholding power to construct a scale-free weighted GCN. The chosen value of β satisfied all the scale-free topology criteria as evidenced in Figure S1. A total of six different color-coded (i.e., green, yellow, turquoise, brown, blue, and grey) co-expression modules were identified by hierarchical clustering and dynamic branch cutting, ranging in size from 39 to 179. To merge modules with highly co-expressed genes, a ME dendrogram was cut at a height of 0.2 corresponding to a correlation of 0.8. Pairwise scatter plots among original MEs and samples representing their degree of correlations between them is shown in Figure 2A. Both yellow and turquoise modules were merged to the brown module based on their high correlation. A total of four meta-modules (i.e., green, blue, brown, and grey) were obtained after merging as compared to the original six modules and are shown in Figure 2B. Since the grey module consisted of unassigned genes, therefore we discarded it for further analysis. Figure 2C shows the GCN heatmap plot depicting TOM among all the genes. Figure 2D–F shows the heatmap plots of three meta-modules along with their corresponding ME levels. Figure S2 shows MM vs. k.in correlation plots of all three meta-modules where all of them are highly significant (based on *p*-value) and correlated (based on Pearson’s correlation coefficient). A total of 151 genes from all the three meta-modules were screened as highly representative considering the threshold (module genes with MM > 0.9).

### 3.3. PPI Network Analysis, Enrichment Analysis, and Hub Genes Identification

**Figure 3:**
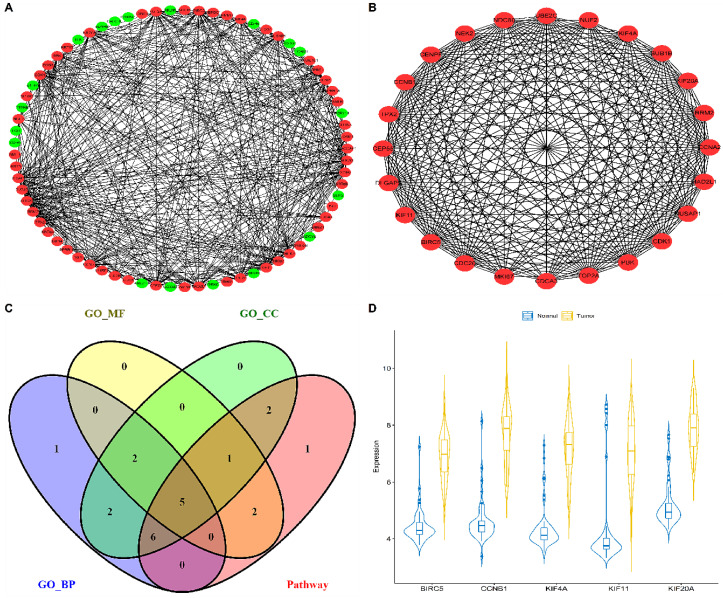
(**A**) Protein–protein interaction (PPI) network comprising 73 nodes and 433 edges constructed using the STRING database. The red and green colored nodes represent up and downregulated proteins. (**B**) Top scoring PPI cluster consists of 24 nodes and 256 edges. **(C)** Overlapping hub genes (BIRC5, CCNB1, KIF4A, KIF11, and KIF20A) between significantly enriched GO terms (BP, MF, and CC) and pathways represented as a Venn plot. (**D**) Violin plots showing the expression distribution density of the 5 hub genes across normal and tumor samples. The top and bottom of the embedded box inside the violin represent the 75th and 25th percentile of the distribution, respectively. The thick black line inside each box represents the median values. Normal and tumor samples are distinguished by blue and yellow colors, respectively. Genes are shown at the bottom and the endpoints of the axis are labelled by the minimum and maximum values.

A total of 148 out of 151 module genes mapped to their corresponding proteins are available in STRING. Total of 73 genes out of them participated in the PPI network at the prescribed confidence score threshold, i.e., >0.9. The constructed PPI network had 73 nodes and 433 edges (Figure 3A), respectively. MCODE plugin revealed a total of four clusters, out of which cluster-1 was selected as the hub cluster since it had the top score (22.261) and involved a total of 24 nodes with 256 edges (Figure 3B), respectively. These 24 top cluster genes were subjected to GO term and pathway enrichment analysis. Lists of all significantly enriched GO terms and pathways can be seen in Supplementary Tables S2 and S3, respectively. A total of five genes were common in all the significantly enriched terms and pathways (Figure 3C) and were considered as the hub genes. Violin plot distribution of these hub genes between tumor and normal samples is shown in Figure 3D. Among all the hub genes, CCNB1 was highly overexpressed in NSCLC samples.

### 3.4. Survival Analysis and Hub Genes Validation

**Figure 4:**
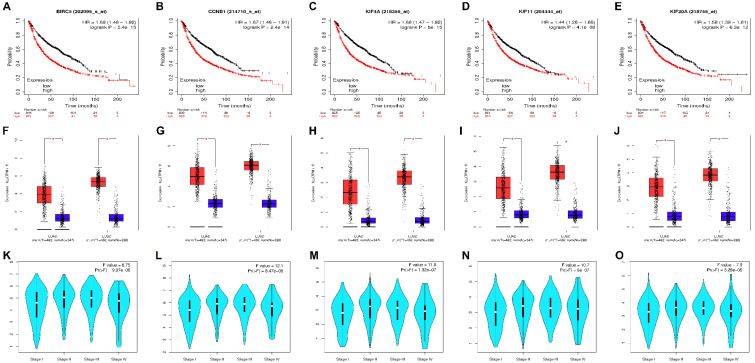
KM survival curves of hub genes **[A]** BIRC5, **[B]** CCNB1, **[C]** KIF4A, **[D]** KIF11, and **[E]** KIF20A plotted using KM plotter. The red line denotes patient samples with a higher gene expression level and the black line denotes patient samples with a lower gene expression level. Higher expression levels of these genes tend to worsen the OS in NSCLC patients. All these genes were highly significant (log rank *p* < 0.05). Boxplots comparing the relative expression levels of hub genes **[F]** BIRC5, **[G]** CCNB1, **[H]** KIF4A, **[I]** KIF11, and **[J]** KIF20A in LUAD and LUSC patients with respect to normal samples. The thick horizontal line in the middle depicts the median, and the lower and upper limits of each box depicts first and third quartiles, respectively. The bottom and top of the error bars depict the minimum and maximum values of expression data. The red and blue boxes depict the NSCLC (LUAD/LUSC) and normal tissues. The method for differential analysis was one-way ANOVA, using disease state as a variable for computing differential expression and asterisk signifies statistically significant with each dot indicating a distinct tumor or normal sample. Violin stage plots of hub genes **[K]** BIRC5, **[L]** CCNB1, **[M]** KIF4A, **[N]** KIF11, and **[O]** KIF20A showing the association of their mRNA expression levels and various tumor stages in NSCLC patients. The white dots and black bars depict the median and interquartile ranges, respectively. The width of the turquoise colored shapes depicts the distribution density. The stages of lung cancer were represented by abscissa and the expression level of each hub genes are represented by its ordinate. The method for differential analysis is one-way ANOVA, using pathological stage as a variable for computing differential expression. A better study fit corresponds to a larger *F*-value. All the genes were statistically significant at *p*-value < 0.05 with CCNB1 having the best study fit (i.e., *F*-value = 12.1).

The KM plotter was used to determine prognostic information of our 5 hub genes (BIRC5, CCNB1, KIF4A, KIF11, KIF20A) to validate the link between their expression levels and metastasis risk in NSCLC. The KM plots of our hub genes shown in Figure 4A–E depicted that higher expression levels of BIRC5 (HR = 1.69; 95% CI = 1.48–1.92; *p* < 0.05), CCNB1 (HR = 1.67; 95% CI = 1.46–1.91; *p* < 0.05), KIF4A (HR = 1.68; 95% CI = 1.47–1.92; *p* < 0.05), KIF11 (HR = 1.44; 95% CI = 1.26–1.65; *p* < 0.05), KIF20A (HR = 1.58; 95% CI = 1.39–1.81; *p* < 0.05) worsened the OS in 1725 NSCLC patients. The median survival time in the high and low expression cohorts of each hub gene is shown in Table 1, respectively.

The results of GEPIA analysis as shown by the boxplots in Figure 4F–J validates that all these five upregulated hub genes (BIRC5, CCNB1, KIF4A, KIF11, KIF20A) were also significantly overexpressed in LUAD and LUSC tissue samples compared with normal tissue samples. Also, GEPIA violin stage plots of hub genes with respect to different pathological staging as shown in Figure 4K–O suggested that their higher expression levels were significantly correlated with advanced TNM stages. Among all the hub genes, BIRC5 and CCNB1 were most overexpressed in LUAD and LUSC samples as evidenced from their boxplots.

### 3.5. Analysis of NSCLC-Specific 3-Node miRNA FFL

**Figure 5:**
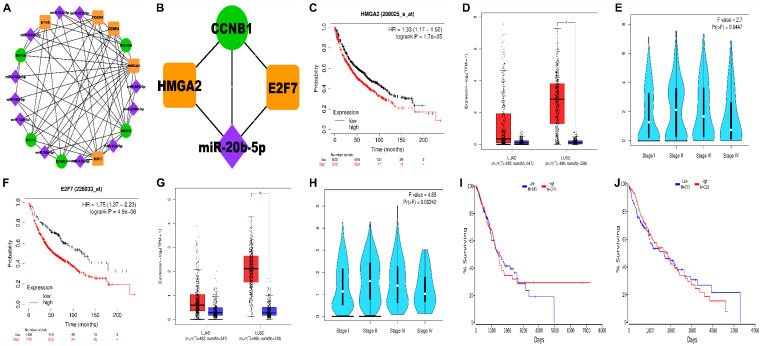
**[A]** NSCLC-specific 3-node miRNA FFL regulatory network comprising 21 nodes and 66 edges, respectively. **[B]** The highest-order network motif consisting of one miRNA (miR-20b-5p), two TFs (HMGA2 and E2F7), and one hub gene (CCNB1), respectively. The green circular nodes represent the hub genes/mRNAs, magenta diamond nodes represent miRNAs, and orange rectangular nodes represent the TFs, respectively. KM survival curves of **[C]** HMGA2 represents a shorter OS in NSCLC patients with its increased expression levels. Boxplot comparing the relative expression levels of **[D]** HMGA2 in LUAD and LUSC patients with respect to normal samples. Violin stage plot of **[E]** HMGA2 showing the association of its expression levels and various tumor stages in NSCLC patient samples. KM survival curves of **[F]** E2F7 represents a shorter OS in NSCLC patients with its increased expression levels. Boxplot comparing the relative expression levels of **[G]** E2F7 in LUAD and LUSC patients with respect to normal samples. Violin stage plot of **[H]** E2F7 showing the association of its expression levels and various tumor stages in NSCLC patient samples. KM survival curves of miR-20b-5p plotted using OncoLnc in **[I]** LUAD and **[J]** LUSC patient samples. Lower expression levels of miR-20b-5p tend to worsen the OS in both LUAD and LUSC samples.

Our 3-node miRNA FFL network (Figure 5A) comprised a total of 21 nodes and 66 edges, respectively.

Among these edges, 19, 25, and 22, belonged to miRNA-mRNA, TF-mRNA, and miRNA-TF interaction pairs, respectively. Table 2 summarizes all the three types of regulatory relationships between miRNAs, TFs, and mRNAs. Among the five hub genes, BIRC5 was targeted by a maximum number of miRNAs (i.e., 8) followed by CCNB1, KIF11, and KIF4A (i.e., 3). miR-20b-5p targeted maximum number of hub genes (i.e., 4).

Among all the TFs, HMGA2 and E2F7 were targeted by a maximum number of miRNAs (i.e., 9 and 7). The analysis of whole FFL revealed that the highest-order network motif (based on the degree) comprised one miRNA (miR-20b-5p), two TFs (HMGA2, E2F7), and one hub gene (CCNB1) and is shown in Figure 5B. KM plots of HMGA2 and E2F7 plotted using KM plotter are shown in Figure 5C,F with the same settings as previously described. The plots indicated that higher expression levels of HMGA2 (HR = 1.33; 95% CI = 1.17–1.52; *p* < 0.05) and E2F7 (HR = 1.75; 95% CI = 1.37–2.23; *p* < 0.05) were associated with shorter OS in NSCLC patients. HMGA2 and E2F7 expression levels were significantly higher in LUSC samples only as evidenced by GEPIA boxplots in Figure 5D,G. Violin stage plots of both HMGA2 and E2F7 were significantly correlated with advanced TNM stages as evidenced from Figure 5E,H. Also, the survival plots of miR-20b-5p in LUAD and LUSC patient samples were plotted using OncoLnc database as shown in Figure 5I–J. The LUAD and LUSC patient samples were split into low and high expression cohorts by assigning lower and higher percentile a 50:50 ratio. Lower expression levels of miR-20b-5p indicated shorter OS in both the LUAD and LUSC patient samples. The median survival time in the high and low expression cohorts of each regulatory element (i.e., HMGA2, E2F7, and miR-20b-5p) is shown in Table 3.

### 3.6. Genomic Alterations of Hub Genes and TFs

**Figure 6:**
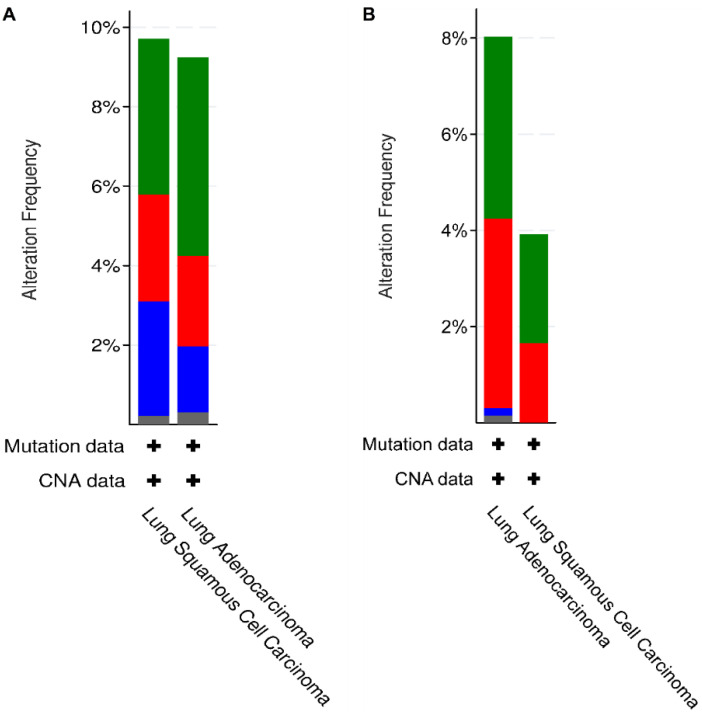
(**A**) Barplot showing the alteration frequency of all hub genes (9.24% of 660 cases of LUAD and 9.71% of 484 cases of LUSC) across TCGA Pan-NSCLC dataset. Green bars represent frequency of mutations (5% in LUAD and 3.93% in LUSC), red bars represent frequency of amplifications (2.27% in LUAD and 2.69% in LUSC), blue bars represent frequency of deep deletion (1.67% in LUAD and 2.89% in LUSC), and grey bars represent frequency of multiple alterations (0.3% in LUAD and 0.2% in LUSC). (**B**) Boxplot showing alteration frequency of 2 TFs—HMGA2 and E2F7—with overall frequency of 6% (8.04% of 660 cases of LUAD and 3.93% of 484 cases of LUSC) in the selected NSCLC dataset. Green bars represent frequency of mutations (3.79% in LUAD and 2.27% in LUSC), red bars represent frequency of amplifications (3.94% in LUAD and 1.65% in LUSC), blue bars represent frequency of deep deletion (0.15% in LUAD), and grey bars represent frequency of multiple alterations (0.15% in LUAD).

cBioportal database was used to scrutinize the specific genetic modifications associated with five hub genes in the selected NSCLC dataset with 1144 samples. Cancer type summary analysis displayed the overall alteration frequency of all hub genes as shown in Figure 6A i.e., 9.24% of 660 cases with a mutation frequency of 5% (33 cases), amplification frequency of 2.27% (15 cases), deep deletion frequency of 1.67% (11 cases), and multiple alterations with a frequency of 0.3% (2 cases) in case of LUAD.

However, in case of LUSC, the overall alteration frequency of all hub genes was 9.71% of 484 cases with a mutation frequency of 3.93% (19 cases), amplification frequency of 2.69% (13 cases), deep deletion frequency of 2.89% (14 cases), and multiple alterations with a frequency of 0.21% (1 case). Moreover, mutation analysis displayed the individual alteration frequencies of hub genes-BIRC5 (0.3%—3 missense mutations), CCNB1 (0.6%—5 missense and 2 truncating mutations), KIF11 (1%—12 missense mutations), KIF20A (0.9%—8 missense and 2 truncating mutations), and KIF4A (2.2%—22 missense and 3 Truncating mutations). Figure 6B shows genetic modification analysis of HMGA2 and E2F7 with an overall gene alteration frequency of both the TFs to be 6% (72 cases out of 1144) which can be further bifurcated into 8.03% of 660 cases of LUAD and 3.93% of 484 cases of LUSC. Moreover, mutation frequency is 3.79% (25 cases) in LUAD and 2.27% (11 cases) in LUSC; amplification frequency of 3.94% (26 cases) for LUAD and 1.65% (8 cases) for LUSC, deep deletion and multiple alteration frequency to be 0.15% respectively (1 case each) for LUAD. Individual mutation analysis of HMGA2 displayed an alteration frequency of 0.4% (5 missense mutations) and E2F7 displayed an alteration frequency of 2.8% (28 missense mutations and 7 truncating mutations).

## 4. Discussion

Lung cancer is a prominent cause of cancer-associated mortality worldwide, with NSCLC being the most frequent type. Insufficient measures for early diagnosis, development of resistance, and delayed prognosis make the available diagnostic and therapeutic tools less effective. Thus, there is an urgent need for the development of more reliable and effective biomarkers as well as therapeutic targets. It has been shown by multifarious studies that non-coding RNAs (ncRNAs) play significant roles in the pathogenesis of different types of cancers and could endeavor a new acumen into the biological aspects of tumorigenesis [38]. Small ncRNAs such as miRNAs have been greatly explored over the past decade. Their regulatory roles in gene expression and other cellular processes have been extensively illustrated but the molecular mechanisms are not fully elucidated. Therefore, a profound exploration of the associated molecular mechanisms is unconditionally important. In consistence, our study was designed to identify the interactions between mRNAs, miRNAs, and TFs taking place in NSCLC, and to further deduce a plausible regulatory network (FFL network) among them.

In this study, we have performed a series of bioinformatics analysis to identify deregulated genes and pathways involved in the development and progression of NSCLC. Upon comparison of DEG profiles of two NSCLC expression datasets retrieved from GEO database, a total of 603 meta-DEGs were screened, out of which 218 were upregulated and 385 were downregulated (Figure 1). WGCNA based scale-free GCN construction gave us three modules in which 151 genes were placed on the basis of their correlation (Figure 2). Later, application of MCODE algorithm to the PPI network consisting of 73 nodes and 433 edges allowed us to identify a hub cluster of genes, consisting of 24 nodes and 256 edges. Afterwards, GO term and pathway enrichment analysis resulted in the identification of five hub genes-BIRC5, CCNB1, KIF11, KIF20A, and KIF4A (Figure 3). The selected hub genes function as a group and may play a momentous role in NSCLC. Pathway enrichment analysis proclaimed that all of these genes were cardinally enriched in cell cycle. In addition, survival analysis of the genes was performed to determine their prognostic significance in terms of overall survival. Further, validation of hub genes determined that all of these were significantly upregulated in lung adenocarcinoma (LUAD) and lung squamous cell carcinoma (LUSC), and consequently associated with advanced TNM stages (Figure 4).

BIRC5 (14.7 kb long) is a mitotic spindle checkpoint gene, present at the telomeric end of chromosome 17. It is a fundamental protein involved in the regulation of mitosis by mediating tumor cell proliferation via β-catenin pathway and tumor cell invasion and migration via TGF-β pathway and PI3K/AKT pathway and apoptosis due to its interaction with DNA damage repair proteins [39] along with several pathological conditions. It has been found to be upregulated in pancreatic cancer, breast cancer, hepatocarcinoma, esophageal carcinoma, and neuroblastoma, with poor clinical outcomes. Our study has highlighted higher expression of BIRC5 (2.02-fold) (Supplementary Table S1) with a shorter OS of NSCLC patients, owing it with the prognosis roles in NSCLC. This gene is critically enriched in cell cycle-related signaling pathways, and hence it becomes logical to assume that the oncogenic functions of BIRC5 are due to these cell cycle mediated mechanisms. However, the mechanism associated with NSCLC remains to be largely unidentified.

Cyclins are a group of proteins that ramble the processes associated with progression and regulation of cell cycle such as cell cycle entry, DNA damage repair and CDK (cyclin-dependent Kinases) mediated control of cell death. CCNB1, CCNB2, and CCNA2 are the authorizing members of the cyclin family, critical for regulation of cellular growth, proliferation, and apoptosis; and consequently, associated with cancer development and progression. They have been found to be highly upregulated in several tumor types including hepatocellular carcinoma. Our study has identified the upregulation (2.6-fold) of CCNB1 in NSCLC tissues as compared to the normal tissues, which has undoubtedly contributed to unfavorable OS in NSCLC patients. CCNB1 is an influential member of the cyclin family. It can initiate mitotic progression by promoting G2/M transition of cells by creating a complex of maturation promoting factor with CDK1, leading to phosphorylation of retinoblastoma (Rb) protein, consequently causing activation of transcription factor E2F2. As a result, E2F2 target genes are activated and regulate G1/S transition. However, E2F2 is lost in most of the cancers, sequentially leading to uncontrolled cell cycle progression. It has been found to be associated with anomalous cellular proliferation in the liver, breast, esophageal, and cervical cancer. We have also found a significant upregulation of CDK1 (1.7-fold), along with CCNB1 in NSCLC (Supplementary Table S1), predicting poor OS of NSCLC patients. Some studies have found CDK1 to be particularly important as it is associated with both OS and relapse-free survival (RFS). Thus, CCNB1 and CDK1 could act as probable prognostic biomarkers of NSCLC. Also, a contemporary study has determined that the levels of anti-CCNB1 antibodies increase with histological grades and stages of cancer, supporting the importance of early-stage screening and recurrence follow-up in advanced stages of lung cancer. Our results are also in concordance with the above studies indicating a high correlation of overexpression of CCNB1 with poor prognosis and clinical stages. Additionally, CCNB1 silencing inhibits cellular proliferation and induces cell senescence and apoptosis via P53 signaling pathway [40].

KIF11 is a mitotic kinesin distributed throughout the cytoplasm and plays a significant role in bipolar mitotic spindle formation. It is also involved in the transport of secretory proteins from the Golgi complex to the surface of non-mitotic cells [41]. The expression of KIF11 is highly noticeable in proliferating cells and tissues during development, however, it is undetectable in case of non-proliferating cells and tissues [42]. It is reported to be an independent prognostic biomarker for early prediction and recurrence intermittence in non-muscle-invasive bladder carcinoma patients and is also correlated with impoverished differentiation of bladder cancer. However, its state of expression and effect on NSCLC remains unclear. Moreover, since it is an important component of mitotic machinery and could be an impressive target of cancer, it becomes coherent to corroborate its role in NSCLC. Bioinformatics analysis in our study has demonstrated a significant correlation between KIF11 upregulation (1.58-fold) (Supplementary Table S1) and progression of NSCLC. A recent study has shown the in vitro and in vivo decrease in cellular proliferation, induction of G2/M cell cycle arrest and increased apoptosis of human breast cancer cells upon RNAi mediated silencing of KIF11. Moreover, our study has found that overexpression of KIF11 is a predictor of poor prognosis and is associated with the advanced stages of NSCLC, indicating its biomarker potential. Some of the studies have demonstrated the mechanism associated with protumorigenic actions of KIF11. They have shown that KIF11 is needed for optimal nascent polypeptide synthesis and is associated with ribosomes during interphase. Thus, upon inhibition of KIF11, ribosomes no longer bind to microtubules, leading to accumulation of polysomes in intact cells and finally resulting in defective elongation or termination during polypeptide synthesis. Moreover, to regulate spindle formation in mitotic cells, KIF11 has been found to be phosphorylated at Thr972 by CDK1, Aurora Kinase A (AURKA), and NIMA-related Kinase 6 (NEK6). However, we have also found the upregulation of CDK1 and AURKA (1.66-fold) (Supplementary Table S1) in NSCLC tissues in our study, suggesting their functional association with KIF11 protein in NSCLC too [41,43].

KIF20A, also known as RAB6KIFL and MKLP2, is a member of Kinesin family-6 and is located on chromosome 5q31.2. It contains a conserved motor domain that generates the energy needed for movement of proteins, upon binding to microtubules and hence is implicated into Golgi apparatus dynamics by interacting with Rab6 small GTPase. It is engaged in mitotic spindle formation and significantly involved in cytokinesis. It is customarily upregulated in malignant tumors, but meagerly expressed in normal tissues except thymus and testis. Its oncogenic properties have been reported in several types of cancers, including hepatocarcinomas, melanomas, pancreatic, and nasopharyngeal carcinomas. It has been found that it is highly upregulated in LUAD and confers malignant phenotype by stimulating cellular proliferation and inhibiting apoptosis. Aneuploidy, abnormal chromosomal distribution, and spindle defects are consequences of aberrant expression of KIF20A and could also be probable causes of tumorigenesis. Our study has found 1.87-fold upregulation of KIF20A (Supplementary Table S1) and its correlation with metastatic status and advanced clinical stage of NSCLC. Consistently, Kaplan-Meier analysis in our study has shown that NSCLC patients with higher expression of KIF20A have a shorter OS and is also associated with clinical stages. In-vitro studies have identified the mechanism by which KIF20A promotes tumorigenesis, i.e., cell cycle dysregulation and inhibition of apoptosis [44]. In addition, cancer cells overexpressing KIF20A can be recognized by host immune system and hence KIF20A-derived peptides can be used as immunotherapeutic agents. They have undergone several clinical trials but there are no data available for NSCLC yet. Studies have also shown that KIF20A knockdown reduced the proliferation, invasion, and migration of NSCLC cells via regulation of JNK signaling pathways [45]. Microarray analysis based study has identified that Forkhead box M1 (FOXM1) positively regulates the expression of KIF20A and upregulation of FOXM1 is associated with several normal and transformed cells of hepatocellular and skin basal cell carcinoma [42]. Consistent with the above findings, we have also observed a significant upregulation (1.9-fold) (Supplementary Table S1) of FOXM1, suggestive of its correlation with KIF20A overexpression in NSCLC.

KIF4A, a microtubule-based motor protein, involved in the regulation of chromosome segregation and spindle formation during mitosis. It is responsible for the generation of directional movements along microtubules and play principal roles in regulation of anaphase spindle dynamics and cytokinesis completion. It has been found to be highly expressed in hepatocellular carcinoma cells and tissues and is also an indicator of poor prognosis. Studies involving siRNA mediated knockdown of KIF4A have resulted in suppression of NSCLC cell growth and its expression promotes cellular invasion. Microarray studies have also demonstrated the decreased OS of NSCLC patients with overexpression of KIF4A [46]. Bioinformatics analysis in our study has determined its upregulation (1.94-fold) in NSCLC patients (Supplementary Table S1). The findings of our study have also indicated the poor OS and association with advanced TNM stage upon overexpression of KIF4A in NSCLC, in consistence with the previous studies. These results are suggestive of the fact that KIF4A is a potential growth factor associated with exceptionally malignant phenotype of lung cancer cells. Despite, the molecular mechanisms behind its overexpression are not yet clarified, it is atypical cancer-testis antigens and scrupulous inhibition of KIF4A using molecular agents could be a promising therapeutic strategy against NSCLC. Nonetheless, one of a recent study has reported contradictory results where the loss of KIF4A lead to multiple mitotic defects such as spindle defects, chromosome misalignment and abnormal cytokinesis, which may lead to tumorigenesis [42]. Thus, extensive research is further required to deduce a useful role of KIF4A in development and progression of NSCLC.

Furthermore, to identify the mutational status of hub genes in NSCLC, we analyzed the information available on cBioPortal tool and found that alteration frequencies were higher in LUAD as compared to LUSC. Individual mutation analysis of hub genes has revealed that KIF4A had the highest alteration frequency of 2.2% in NSCLC (Figure 6). This is indicative of the association of genetic modifications in deregulated cellular processes, which could be a significant cause of upregulation of these genes and hence aberrations in pathways associated with the genes. Moreover, a deeper understanding of associations between somatic mutations and cancer traits would be of great help in designing precision cancer therapy.

Besides this, to expedite the elucidation of regulatory network involved in NSCLC, a 3-node miRNA FFL was generated, consisting of a miRNA (miR-20b-5p), a hub gene (CCNB1), and two transcription factors (HMGA2 and E2F7) (Figure 5). miR-20-5p is present at a site (human chromosome Xq26.2) that has been reported to be related to the development and progression of a number of cancer types. It is proclaimed to exert context-dependent tumor-suppressive or oncogenic roles affecting cellular proliferation, migration, and apoptosis. It is downregulated in renal cell carcinoma but upregulated in breast cancer and NSCLC. However, its biological functions in NSCLC are largely unclear [47]. HMGA2 and E2F7 are the two direct targets of miR-20b-5p. HMGA proteins are non-histone nuclear proteins, commonly recognized as architectural transcription factors as they are involved in assemblage of gene transcription-associated multiprotein complexes. Besides chromatin organization of the transcription machinery in a structure that concedes gene transcription, it also interacts with minor grooves of several AT-rich promoters and enhancers to exert transcriptional activity. There are four types of HMGA proteins-HMGA1a, HMGA1b, HMGA1c, and HMGA2. HMGA2 is associated with human malignant tumors and neoplastic transformation of thyroid cells [48]. It regulates cellular proliferation and metastasis in lung cancer. Notably, both HMGA1 and HMGA2 are actively involved in the construction of senescence-associated heterochromatin and prolongation of growth-arrested state. HMGA2 is customarily rearranged in benign tumors of mesenchymal origin. Chromosomal aberrations in the region 12q14-15, affecting HMGA2 are generally observed in a wide range of tumors [49]. It is highly upregulated in majority of human prolactinomas with genetic alterations such as chromosome 12 trisomy and tetrasomy. Our study has identified the upregulation of HMGA2 in NSCLC, which is correlated with poor OS and advanced clinical stage (Figure 5). HMGA2 is also associated with transformation of NSCLC cells but the associated mechanisms are still unknown. HMGA2 is reported to be involved in pRb pathway. pRb gene is related to progression of cell cycle via E2F family of transcription factors. They have been well defined as G1/S regulators. HMGA2 overexpression enhances the transcriptional activity of E2F1 proteins. These proteins are well documented to promote transcription of genes involved in S phase of the cell cycle. Prior to the entry of cells into S phase, cyclin CCNB1 forms a complex with CDK1, which phosphorylates Rb at multiples sites, leading to its inactivation and activation of E2F1/E2F2, both of which are involved in the regulation of G1/S transition. However, E2F2 is mostly lost in several tumors, but E2F1 is still activated leading to the transcriptional activation of its target genes. On the contrary, pRb recruits HDAC-1 (Histone deacetylase 1) which removes acetyl groups to repress the transcription. But HMGA2 binds to pRb and displaces HDAC1 so that E2F1 is continually activated and resumes the transcription of its target genes [48]. E2F1 is an effective stimulant of apoptosis and hence keeps a regulation of cells entering into S phase. However, continuous activation of E2F1 may also cause the transcriptional activation of E2F7 (a transcriptional target of E2F1 and also exerts poor OS (Figure 5)). E2F7 is highly upregulated in case of NSCLC, which, in turn, exerts a negative feedback on E2F1, causing its transcriptional repression and hence represses the apoptosis function of E2F1 and aberrantly allow the cells to enter into S phase of cell cycle [50]. Moreover, genetic modification analysis of both HMGA2 and E2F7 has displayed an overall alteration frequency of 8.03% in LUAD and 3.93% in LUSC (Figure 6). This is suggestive of the fact that epigenetic mechanisms could be an important factor in upregulation of these transcription factors. Overall, it can be speculated that genetic modifications could lead to an abrupt upregulation of HMGA2 and E2F7, which are involved in uncontrolled transition of the cells from G1 to S phase via CCNB1 and CDK1 mediated phosphorylation of Rb protein. pRb protein causes release of E2F1 proteins, which upon uncontrolled activation causes activation of E2F7 proteins, which acts as a novel target to mediate repression of E2F1-associated apoptosis and hence removes the regulatory powers of E2F1, leading to continued proliferation of NSCLC cells.

Furthermore, downregulation of miR-20b-5p worsens the OS of NSCLC patients, as demonstrated by Kaplan Meir Survival analysis (Figure 5), which is suggestive of the tumor-suppressive functions of miR-20b-5p. A recent study has shown that 3′UTR of HMGA2 contains let-7 binding sites and chromosomal aberrations in HMGA2 may lead to loss of miRNA-mediated repression and hence upregulation of HMGA2 [49]. Similarly, our study has found that mir-20b-5p targets both HMGA2 and E2F7 (both having a good frequency of genetic alterations in NSCLC, particularly in LUAD) and genetic alterations in HMGA2 and E2F7 may alter its 3′UTR site, consequently inhibiting miR-20b-5p mediated repression of HMGA2 and E2F7. Moreover, studies have also reported that oxidative stress, which is an important aspect of tumor microenvironment, downregulates miR-20b-5p and E2F1 via overexpression of HMGA2 [51]. This can be explained as, oxidative damage causes activation of HMGA2 which participates in ATM/CHK-mediated DNA damage repair systems causing prolonged phosphorylation of CHK1, facilitating DNA repair, cell cycle delay in G2/M phase until the DNA is fully repaired and increases survival and chemoresistance of cancer cells. Thus, HMGA2 exerts oncogenic functions via E2F7-mediated repression of pro-apoptotic E2F1 and oxidative damage-mediated cell cycle delay in G2/M phase and development of chemoresistance. Therefore, upon overexpression of miR-20b-5p, the tumor-suppressive effects could be rescued back by regulating cell cycle and promoting apoptosis, which could prove to be an effective strategy of treatment of NSCLC.

In addition, studies have identified that overexpression of HMGA2 also affects direct NF-κβ targets, most of which are involved in cytokine signaling such as IL6, IL1R1, IL11, IL15, and LIF1. Genes in CXC chemokine cluster in chromosome band 4q13.3, such as CXCL6 are highly downregulated but CXCL12 is upregulated [49]. Our study has identified the downregulation of IL6 (2.20-fold), IL1R1 (1.73-fold), CXCR2 (2.20-fold), CXCL3 (1.77-fold) and an upregulation of CXCL13 (2.78-fold) and CXCL14 (2.17-fold) (Supplementary Table S1). Downregulation of these chemokines are associated with the escape from host immune response by reducing the attraction of neutrophils, leukocytes, and activation of adaptive immune response. Moreover, upregulation of CXCL13 and CXCL14 are associated with a reduction in HLA-DRA and MHC-Class II, because of which cancer-specific antigens are not presented by dendritic cells and hence cancer cells evade immunosurveillance [52]. Thus, HMGA2 also contribute in evading host immune response and continue cell proliferation, providing a survival advantage to cancer cells.

Overall, this study has identified certain novel molecules (miR-20b-5p, CCNB1, HMGA2, and E2F7), all of which could possess a great therapeutic and prognostic potential, which has not yet been identified in NSCLC. Studies have reported that these targets are commonly involved in the deregulation of cell cycle because of genetic modifications, and escape from immunosurveillance, which are predominant hallmarks of cancer. Our study has also indicated their associative roles in cell cycle deregulation and immunosurveillance, thus these genes, when further explored could prove to be milestones in lung cancer research and could be very useful in designing personalized therapies against cancer. Moreover, it is a proposed mechanism, suggested by the network obtained from bioinformatics analysis, however, extensive in vitro and in vivo research is further required to validate the above processes.

## 5. Conclusions

In conclusion, for NSCLC, all the hub genes—BIRC5, CCNB1, KIF11, KIF20A, and KIF4A, TFs—HMGA2 and E2F7 are associated with poor OS and advanced stages of clinical disease. They may serve as assuring prognostic predictors and therapeutic targets. The upregulation of these genes may facilitate the activation of cell cycle-associated pathways to take part in the development of NSCLC. However, upregulation of CCNB1, along with CDK1, forms a complex that participates in pRb pathway of cell cycle regulation, wherein HMGA2 and E2F7 are actively involved. Moreover, genetic modification status of HMGA2 and E2F7 determines the involvement of epigenetic modifications in affecting miR-20b-5p mediated repression of these TFs, sequentially resulting in their upregulation. Upregulation of HMGA2 participates in several mechanisms such as E2F7-mediated repression of pro-apoptotic effects of E2F1, cell cycle delay until the DNA damage is repaired, cancer cell survival, and development of chemoresistance, that confer survival-specific and evasion of immune surveillance (via downregulation of IL6, IL1R1 and upregulation of chemokines such as CXCL13 and CXCL14) advantage to cancer cells. Our findings contribute in providing an innovative comprehension into NSCLC via miR-20b-5p/CCNB1/HMGA2/E2F7. Nonetheless, cancer is an outcome of complex molecular processes, and hence extensive experimental corroborations are needed to validate the cell cycle and immune system-related signaling for NSCLC.

## Figures and Tables

**Table 1:** Median survival time in different expression cohorts with respect to each hub gene.

Gene	Low Expression Cohort (Months)	High Expression Cohort (Months)
BIRC5	92.6	42
CCNB1	89	42
KIF4A	89	40.2
KIF11	79.54	45.27
KIF20A	91	43.83

**Table 2:** Summary of regulatory relationships between NSCLC-associated DEGs, miRNAs, and TFs.

Relationship	No. of Edges	No. of miRNAs	No. of Genes	No. of TFs
miRNA-gene ^a^	19	11	5	-
TF-gene ^b^	25	-	5	5
miRNA-TF ^c^	22	11	-	5

^a^ miRNA-gene: miRNA repression of genes; ^b^ TF-gene: TF regulation of genes; ^c^ miRNA-TF: miRNA repression of TFs.

**Table 3:** Median survival time in different expression cohorts with respect to miRNA (miR-20b-5p) and TFs (HMGA2, E2F7).

Gene	Low Expression Cohort (Months)	High Expression Cohort (Months)
miR-20b-5p (LUAD)	21.43	22.55
miR-20b-5p (LUSC)	22.17	26.53
HMGA2	74.18	52
E2F7	119.87	57

## Supplementary Materials

The following are available online at https://www.mdpi.com/2079-7737/9/8/201/s1. Table S1: List of meta-DEGs; Table S2: List of significantly enriched GO terms for the identified PPI cluster genes; Table S3: List of significantly enriched pathways for the identified PPI cluster genes; Figure S1: [A] Analysis of scale-free fitting indices (R^2) for various possible soft-thresholding powers (β). [B] Analysis of mean connectivity for various possible soft-thresholding powers (β). [C] Histogram of network connectivity distribution when β = 16. [D] 〖log〗_10 (k) vs 〖log〗_10 (p(k)) plot of the same histogram where the scale-free topology is depicted by the approximate straight line relationship (high R^2 = 0.80) and a negative value of slope (slope = −1.32). Figure S2. Correlation plots of k.in (x-axis) vs. MM (y-axis) for [A] brown, [B] blue, and [C] green modules, respectively. The color indicates the module and the dots indicate the genes within that module. After raising the MM to β, it is highly correlated with k.in for all the modules.

## References

[1] Herbst R.S., Heymach J.V., Lippman S.M. (2008). Lung cancer. N. Engl. J. Med..

[2] Oser M.G., Niederst M.J., Sequist L.V., Engelman J.A. (2015). Transformation from non-small-cell lung cancer to small-cell lung cancer: Molecular drivers and cells of origin. Lancet Oncol..

[3] Molina J.R., Yang P., Cassivi S.D., Schild S.E., Adjei A.A. (2008). Non-small cell lung cancer: Epidemiology, risk factors, treatment, and survivorship. Mayo Clin. Proc..

[4] Wang H., Shen Q., Zhang X., Yang C., Cui S., Sun Y., Wang L., Fan X., Xu S. (2017). The Long Non-Coding RNA XIST Controls Non-Small Cell Lung Cancer Proliferation and Invasion by Modulating miR-186-5p. Cell. Physiol. Biochem..

[5] Lee R.C., Feinbaum R.L., Ambros V. (1993). The *C. elegans* heterochronic gene lin-4 encodes small RNAs with antisense complementarity to lin-14. Cell.

[6] Ambros V., Bartel B., Bartel D.P., Burge C.B., Carrington J.C., Chen X., Dreyfuss G., Eddy S.R., Griffiths-Jones S., Marshall M. (2003). A uniform system for microRNA annotation. RNA N. Y. N.

[7] Jin P., Zarnescu D.C., Ceman S., Nakamoto M., Mowrey J., Jongens T.A., Nelson D.L., Moses K., Warren S.T. (2004). Biochemical and genetic interaction between the fragile X mental retardation protein and the microRNA pathway. Nat. Neurosci..

[8] Lee Y.S., Dutta A. (2009). MicroRNAs in cancer. Annu. Rev. Pathol..

[9] Liu X., Sempere L.F., Ouyang H., Memoli V.A., Andrew A.S., Luo Y., Demidenko E., Korc M., Shi W., Preis M. (2010). MicroRNA-31 functions as an oncogenic microRNA in mouse and human lung cancer cells by repressing specific tumor suppressors. J. Clin. Investig..

[10] Alexiou P., Maragkakis M., Papadopoulos G.L., Reczko M., Hatzigeorgiou A.G. (2009). Lost in translation: An assessment and perspective for computational microRNA target identification. Bioinforma. Oxf. Engl..

[11] Holbro T., Beerli R.R., Maurer F., Koziczak M., Barbas C.F., Hynes N.E. (2003). The ErbB2/ErbB3 heterodimer functions as an oncogenic unit: ErbB2 requires ErbB3 to drive breast tumor cell proliferation. Proc. Natl. Acad. Sci. USA.

[12] Kumar M.S., Erkeland S.J., Pester R.E., Chen C.Y., Ebert M.S., Sharp P.A., Jacks T. (2008). Suppression of non-small cell lung tumor development by the let-7 microRNA family. Proc. Natl. Acad. Sci. USA.

[13] Sampson V.B., Rong N.H., Han J., Yang Q., Aris V., Soteropoulos P., Petrelli N.J., Dunn S.P., Krueger L.J. (2007). MicroRNA let-7a down-regulates MYC and reverts MYC-induced growth in Burkitt lymphoma cells. Cancer Res..

[14] Mayr C., Hemann M.T., Bartel D.P. (2007). Disrupting the pairing between let-7 and Hmga2 enhances oncogenic transformation. Science.

[15] Lee Y.S., Dutta A. (2007). The tumor suppressor microRNA let-7 represses the HMGA2 oncogene. Genes Dev..

[16] Fabbri M., Garzon R., Cimmino A., Liu Z., Zanesi N., Callegari E., Liu S., Alder H., Costinean S., Fernandez-Cymering C. (2007). MicroRNA-29 family reverts aberrant methylation in lung cancer by targeting DNA methyltransferases 3A and 3B. Proc. Natl. Acad. Sci. USA.

[17] Yanaihara N., Caplen N., Bowman E., Seike M., Kumamoto K., Yi M., Stephens R.M., Okamoto A., Yokota J., Tanaka T. (2006). Unique microRNA molecular profiles in lung cancer diagnosis and prognosis. Cancer Cell.

[18] Cummins J.M., He Y., Leary R.J., Pagliarini R., Diaz L.A., Sjoblom T., Barad O., Bentwich Z., Szafranska A.E., Labourier E. (2006). The colorectal microRNAome. Proc. Natl. Acad. Sci. USA.

[19] Clough E., Barrett T. (2016). The Gene Expression Omnibus Database. Methods Mol. Biol. Clifton N. J..

[20] Singh P., Rai A., Dohare R., Arora S., Ali S., Parveen S., Syed M.A. (2020). Network-based identification of signature genes KLF6 and SPOCK1 associated with oral submucous fibrosis. Mol. Clin. Oncol..

[21] Shriwash N., Singh P., Arora S., Ali S.M., Ali S., Dohare R. (2019). Identification of differentially expressed genes in small and non-small cell lung cancer based on meta-analysis of mRNA. Heliyon.

[22] Benjamini Y., Hochberg Y. (1995). Controlling the False Discovery Rate: A Practical and Powerful Approach to Multiple Testing. J. R. Stat. Soc. Ser. B Methodol..

[23] Ahmad S., Singh P., Sharma A., Arora S., Shriwash N., Rahmani A.H., Almatroodi S.A., Manda K., Dohare R., Syed M.A. (2019). Transcriptome Meta-Analysis Deciphers a Dysregulation in Immune Response-Associated Gene Signatures during Sepsis. Genes.

[24] Langfelder P., Horvath S. (2008). WGCNA: An R package for weighted correlation network analysis. BMC Bioinformatics.

[25] Zhang B., Horvath S. (2005). A general framework for weighted gene co-expression network analysis. Stat. Appl. Genet. Mol. Biol..

[26] Cai Y., Mei J., Xiao Z., Xu B., Jiang X., Zhang Y., Zhu Y. (2019). Identification of five hub genes as monitoring biomarkers for breast cancer metastasis in silico. Hereditas.

[27] Szklarczyk D., Gable A.L., Lyon D., Junge A., Wyder S., Huerta-Cepas J., Simonovic M., Doncheva N.T., Morris J.H., Bork P. (2019). STRING v11: Protein–protein association networks with increased coverage, supporting functional discovery in genome-wide experimental datasets. Nucleic Acids Res..

[28] Shannon P., Markiel A., Ozier O., Baliga N.S., Wang J.T., Ramage D., Amin N., Schwikowski B., Ideker T. (2003). Cytoscape: A software environment for integrated models of biomolecular interaction networks. Genome Res..

[29] Di Y., Chen D., Yu W., Yan L. (2019). Bladder cancer stage-associated hub genes revealed by WGCNA co-expression network analysis. Hereditas.

[30] Kuleshov M.V., Jones M.R., Rouillard A.D., Fernandez N.F., Duan Q., Wang Z., Koplev S., Jenkins S.L., Jagodnik K.M., Lachmann A. (2016). Enrichr: A comprehensive gene set enrichment analysis web server 2016 update. Nucleic Acids Res..

[31] Győrffy B., Surowiak P., Budczies J., Lánczky A. (2013). Online survival analysis software to assess the prognostic value of biomarkers using transcriptomic data in non-small-cell lung cancer. PLoS ONE.

[32] Tang Z., Kang B., Li C., Chen T., Zhang Z. (2019). GEPIA2: An enhanced web server for large-scale expression profiling and interactive analysis. Nucleic Acids Res..

[33] Sticht C., De La Torre C., Parveen A., Gretz N. (2018). miRWalk: An online resource for prediction of microRNA binding sites. PLoS ONE.

[34] Li J.-H., Liu S., Zhou H., Qu L.-H., Yang J.-H. (2014). starBase v2.0: Decoding miRNA-ceRNA, miRNA-ncRNA and protein–RNA interaction networks from large-scale CLIP-Seq data. Nucleic Acids Res..

[35] Keenan A.B., Torre D., Lachmann A., Leong A.K., Wojciechowicz M.L., Utti V., Jagodnik K.M., Kropiwnicki E., Wang Z., Ma’ayan A. (2019). ChEA3: Transcription factor enrichment analysis by orthogonal omics integration. Nucleic Acids Res..

[36] Cerami E., Gao J., Dogrusoz U., Gross B.E., Sumer S.O., Aksoy B.A., Jacobsen A., Byrne C.J., Heuer M.L., Larsson E. (2012). The cBio cancer genomics portal: An open platform for exploring multidimensional cancer genomics data. Cancer Discov..

[37] Campbell J.D., Alexandrov A., Kim J., Wala J., Berger A.H., Pedamallu C.S., Shukla S.A., Guo G., Brooks A.N., Murray B.A. (2016). Distinct patterns of somatic genome alterations in lung adenocarcinomas and squamous cell carcinomas. Nat. Genet..

[38] Iqbal M.A., Arora S., Prakasam G., Calin G.A., Syed M.A. (2019). MicroRNA in lung cancer: Role, mechanisms, pathways and therapeutic relevance. Mol. Aspects Med..

[39] Cao Y., Zhu W., Chen W., Wu J., Hou G., Li Y. (2019). Prognostic Value of BIRC5 in Lung Adenocarcinoma Lacking EGFR, KRAS, and ALK Mutations by Integrated Bioinformatics Analysis. Dis. Markers.

[40] Zhang H., Zhang X., Li X., Meng W.-B., Bai Z.-T., Rui S.-Z., Wang Z.-F., Zhou W.-C., Jin X.-D. (2018). Effect of CCNB1 silencing on cell cycle, senescence, and apoptosis through the p53 signaling pathway in pancreatic cancer. J. Cell. Physiol..

[41] Daigo K., Takano A., Thang P.M., Yoshitake Y., Shinohara M., Tohnai I., Murakami Y., Maegawa J., Daigo Y. (2018). Characterization of KIF11 as a novel prognostic biomarker and therapeutic target for oral cancer. Int. J. Oncol..

[42] Yu Y., Feng Y.-M. (2010). The role of kinesin family proteins in tumorigenesis and progression: Potential biomarkers and molecular targets for cancer therapy. Cancer.

[43] Pei Y.-Y., Li G.-C., Ran J., Wei F.-X. (2017). Kinesin family member 11 contributes to the progression and prognosis of human breast cancer. Oncol. Lett..

[44] Zhao X., Zhou L.-L., Li X., Ni J., Chen P., Ma R., Wu J., Feng J. (2018). Overexpression of KIF20A confers malignant phenotype of lung adenocarcinoma by promoting cell proliferation and inhibiting apoptosis. Cancer Med..

[45] Xie F., He C., Gao S., Yang Z., Li L., Qiao L., Fang L. (2020). KIF20A silence inhibits the migration, invasion and proliferation of non-small cell lung cancer and regulates the JNK pathway. Clin. Exp. Pharmacol. Physiol..

[46] Taniwaki M., Takano A., Ishikawa N., Yasui W., Inai K., Nishimura H., Tsuchiya E., Kohno N., Nakamura Y., Daigo Y. (2007). Activation of KIF4A as a prognostic biomarker and therapeutic target for lung cancer. Clin. Cancer Res..

[47] Peng L., Li S., Li Y., Wan M., Fang X., Zhao Y., Zuo W., Long D., Xuan Y. (2019). Regulation of BTG3 by microRNA-20b-5p in non-small cell lung cancer. Oncol. Lett..

[48] Fedele M., Visone R., De Martino I., Troncone G., Palmieri D., Battista S., Ciarmiello A., Pallante P., Arra C., Melillo R.M. (2006). HMGA2 induces pituitary tumorigenesis by enhancing E2F1 activity. Cancer Cell.

[49] Henriksen J., Stabell M., Meza-Zepeda L.A., Lauvrak S.A., Kassem M., Myklebost O. (2010). Identification of target genes for wild type and truncated HMGA2 in mesenchymal stem-like cells. BMC Cancer.

[50] Moon N.-S., Dyson N. (2008). E2F7 and E2F8 keep the E2F family in balance. Dev. Cell.

[51] Tai L., Huang C.-J., Choo K.B., Cheong S.K., Kamarul T. (2020). Oxidative Stress Down-Regulates MiR-20b-5p, MiR-106a-5p and E2F1 Expression to Suppress the G1/S Transition of the Cell Cycle in Multipotent Stromal Cells. Int. J. Med. Sci..

[52] Mukaida N., Sasaki S., Baba T. (2014). Chemokines in cancer development and progression and their potential as targeting molecules for cancer treatment. Mediat. Inflamm..

