# Supplementary material for: Unravelling the Role of miR-20b-5p, CCNB1, HMGA2 and E2F7 in Development and Progression of Non-Small Cell Lung Cancer (NSCLC)
Source: Biology (Basel). 2020 Aug 1;9(8):201. doi: 10.3390/biology9080201 (PMC7465122; doi:10.3390/biology9080201)
Supplement: Supplementary file 1 [file biology-09-00201-s001.zip › Supp_R1/Supplementary Figure Legends.docx]

**Supplementary Figure Legends**

**Figure S1. [A]** Analysis of scale-free fitting indices ($\boldsymbol{R}^{\boldsymbol{2}}$) for various possible soft-thresholding powers ($\boldsymbol{\beta}$). **[B]** Analysis of mean connectivity for various possible soft-thresholding powers ($\boldsymbol{\beta}$). **[C]** Histogram of network connectivity distribution when $\boldsymbol{\beta=16}$. **[D]** $\boldsymbol{log}_{\boldsymbol{10}}\left( \boldsymbol{k} \right)$ vs $\boldsymbol{log}_{\boldsymbol{10}}\left( \boldsymbol{p(k)} \right)$ plot of the same histogram where the scale-free topology is depicted by the approximate straight line relationship (high $\boldsymbol{R}^{\boldsymbol{2}}\boldsymbol{=0.80}$) and a negative value of slope $(\boldsymbol{slope=-1.32}$).

**Figure S2.** Correlation plots of $\boldsymbol{k.in}$ (x-axis) vs $\boldsymbol{MM}$ (y-axis) for **[A]** brown, **[B]** blue, and **[C]** green modules, respectively. The color indicates the module and the dots indicate the genes within that module. After raising the $\boldsymbol{MM}$ to $\boldsymbol{\beta}$, it is highly correlated with $\boldsymbol{k.in}$ for all the modules.
